# A putative species complex in the Sea of Japan revealed by DNA sequence data: a study on *Lottia* cf. *kogamogai* (Gastropoda: Patellogastropoda)

**DOI:** 10.1111/jzs.12120

**Published:** 2016-01-08

**Authors:** Alen Kristof, André L. de Oliveira, Konstantin G. Kolbin, Andreas Wanninger

**Affiliations:** ^1^ Department of Integrative ZoologyUniversity of ViennaViennaAustria; ^2^ Laboratory of Cell DifferentiationA.V. Zhirmunsky Institute for Marine BiologyFar East Branch of the Russian Academy of SciencesVladivostokRussia

**Keywords:** Sympatric distribution, Peter the Great Bay, water currents, speciation

## Abstract

A putative new limpet species (Patellogastropoda) from the Sea of Japan is revealed by molecular genetic analyses using the mitochondrial markers *16S* rRNA and *cytochrome c oxidase* subunit I (*CO1*), as well as the DNA marker *18S* rRNA. Our data indicate that the limpet, collected in the Peter the Great Bay (Russian Federation), is not, as its morphology suggests, the Japanese species *Lottia kogamogai* Sasaki and Okutani, 1994, and might also hint towards another putative species complex in the Sea of Japan. The different currents between the Far East Asian mainland (cold, subpolar jet running southwards) and the Japanese archipelago (warm, subtropical jet running northwards) are likely to act as a barrier that has a substantial influence on species distribution in these waters. Accordingly, our results indicate that it is about time for a revision of patellogastropod species with a reported distribution in Japanese and Far Eastern Russian waters by an integrative approach using molecular genetic and morphological characters. The species investigated herein is referred to as *Lottia* cf. *kogamogai* until it is morphologically re‐examined and compared with primary type specimens of known species.

Patellogastropods, also called ‘true limpets’, are well‐known members of intertidal rocky seashore communities throughout all oceans (Underwood 2000; Range et al. 2008; Miloslavich et al. 2013). These animals exclusively exhibit a cap‐like shell, range in size from few millimetres to over 20 cm, and are grazers that feed on algae, seagrass, wood or detritus (Lindberg 2008). In general, species are identified and delineated from each other by some external (shell shape, sculpture, colour, apex position, colour of the foot and tentacles) and internal (radular sac, radula) morphological characters (Espoz et al. 2004; Chernyshev and Chernova 2007; Hoffman et al. 2011). A sympatric distribution of different patellogastropod species at the same locality is often observed (Flores‐Garza et al. 2011; Flores‐Rodríguez et al. 2014) as well as a high phenotypic intraspecific variability in the same species among different localities, which strongly depend on environmental conditions (Jobe 1968; Lindberg 1979; Simpson 1985). This variability in important diagnostic characters such as the shell or the radula complicates species recognition. Furthermore, hybridization between limpet species has been reported to occur and thus, it is an another issue that has to be taken into account (Weber and Hawkins 2002; De Aranzamendi et al. 2009). As a result, numerous superficially similar species have been grouped together, leading to species complexes with numerous subspecies and a wide or even cosmopolitan distribution. Some of these species complexes in Patellogastropoda have been revised by the use of multiple morphological and molecular genetic characters (Sasaki and Okutani 1994; Simison and Lindberg 1999; Nakano and Ozawa 2005; Reisser et al. 2012).

At present, molecular genetic approaches are widely used in order to complement morphological studies and to solve problems in species identification. Thereby, DNA sequence data are a widely used molecular genetic tool for species identification and classification that is based on standardised and relatively short (400–800 bp) DNA sequences (Hebert et al. 2003). A gene fragment of the mitochondrial *cytochrome c oxidase* subunit I (*CO1*) has been established as the barcoding gene in animals (Kress and Erickson 2008). The suitability of the *CO1* gene for species identification in patellogastropods has been demonstrated in a number of recent studies (Mauro et al. 2003; Lin et al. 2015). However, species identification based on a single character – regardless whether morphological or molecular – poses not only a risk for misidentifications but might also lead to taxonomic inflation and may even raise conservation issues (Wheeler 2005; Zachos 2015). By contrast, studies that include multiple genes have proven useful in solving identification issues, often revealing cryptic species (Nakano and Ozawa 2005; Espinosa and Ozawa 2006; De Aranzamendi et al. 2009; Yu et al. 2014; Harasewych 2015).

The aim of this study was to include widely used genetic markers (i.e. nuclear *18S* rRNA, mitochondrial *16S* rRNA and *CO1*) that would not only supplement the morphological identification made during field sampling but also provide reference sequences that might be used in future studies on this limpet. So far, 17 different patellogastropod species are known from the Peter the Great Bay (Sea of Japan, Russian Federation), of which up to four different species [*Lottia* cf. *kogamogai*,* Lottia versicolor* (Moskalev in Golikov and Scarlato, 1967), *Lottia tenuisculpta* Sasaki and Okutani 1994; *Nipponacmea moskalevi* Chernyshev and Chernova, 2002] co‐occur at the sampling site (Vostok Bay; Fig. 1; N 42°53′35.5″, E 132°44′00.8″) that are thought to be identified by their shell morphology (Chernyshev and Chernova 2007; Gulbin 2010). For the purpose of developmental, morphological and molecular gene‐expression studies, adult specimens of *L. *cf. *kogamogai* were sampled from July until August 2011, and 2013 from intertidal rocks and stones in the vicinity of the marine biological station ‘Vostok’, cultured and fixed in different solutions (see Kristof et al. 2015, for details).

**Figure 1:**
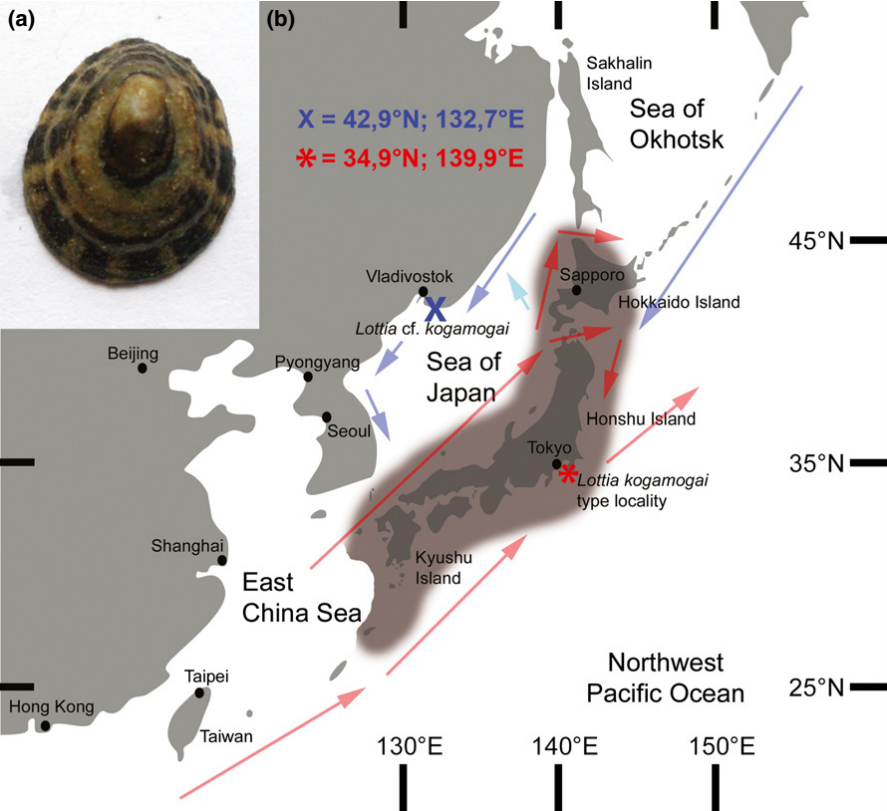
Adult specimen of the patellogastropod investigated herein (a) and map of the sampling site (b). (a) *Lottia* cf. *kogamogai*, shell length approximately 5 cm, dorsal view with anterior facing upwards. (b) Geographical distribution of *L. kogamogai* Sasaki and Okutani 1994 along the Japanese Archipelago (grey cloud) with its type locality (Banda, Tateyama, Boso Peninsula, Japan; red asterisk). The sampling site of *L. *cf. *kogamogai* is indicated by the blue X. Arrows represent widely recognized relatively warm (red), temperate (cyan) and cold (blue) ocean currents. Map and currents were redrawn and modified after Sasaki and Okutani (1994) and Moors et al. (2006).

Adult voucher specimens and additional material are deposited at the Natural History Museum in Vienna (NHMW; accession number Mollusca NHMW 110180) and at the Department of Integrative Zoology, University of Vienna (UV).

The three above mentioned genetic markers (*18S*,* 16S* and *CO1*) have been retrieved from an existing transcriptome library that was generated from *L*. cf. *kogamogai* mRNA mainly for molecular gene‐expression studies on this species with a 454 FLX sequencer (Eurofins, MWG, Ebersberg, Germany).

Several hundred *L. *cf. *kogamogai* embryos, larvae and juveniles of key developmental stages (i.e. trochophore, veliger, pediveliger, metamorphic competent, first juvenile stages), as well as 15 adult specimens, were separately stored in RNA*later* (Sigma, St. Louis, MO, USA). Total RNA was extracted from each developmental stage as well as different adult tissues using TRIZOL (Sigma) and miRCURY RNA isolation kit (Exiqon, Vedbaek, Denmark). A mixed mRNA sample was used for cDNA library preparation, whereby two‐thirds derived from the different developmental stages and one‐third from adult tissues. This sequencing produced 402.814 raw reads with an average length of 639 bases.

The raw reads were trimmed (removal of adaptor sequences) with the program sff_extract present in the seq_crumbs package (http://bioinf.comav.upv.es/seq_crumbs/), a collection of small sequence processing utilities. The filtered transcriptomic dataset was then reconstructed into contiguous cDNA sequences using the mira assembler v4.0 (Chevreux et al. 2004). The assembly process reconstructed 27 737 707 bp, located in a total of 34 794 contigs, with N50 value of 817 bp. In order to perform the DNA sequence analysis, similarity searches were carried out between the assembled dataset and classical genetic markers (*18S* rRNA, *16S* rRNA and *CO1*). Sequences of these three genes were identified within the transcriptome library of *L. *cf. *kogamogai* (GenBank accession numbers: KU053948, KU053949 and KU053950), and additional patellogastropod gene sequences were downloaded from GenBank as well as sequences of *L. kogamogai* Sasaki and Okutani 1994; and two other molluscs (Table S1). Each *L. *cf. *kogamogai* gene was independently aligned together with their respective homologues using the programme mafft v7.123b (Katoh and Standley 2013). The multiple sequence alignments were individually analysed and manually edited with the program aliview (Larsson 2014). The phylogenetic analyses were conducted using beast2 (Bouckaert et al. 2014) with different datasets consisting of the nuclear *18S* rRNA gene, and the mitochondrial genes *16S* rRNA and *CO1* individually (Figure S1) as well as in combination (Fig. 2). The best substitution model was determined using AIC as implemented in jmodetest2 (Darriba et al. 2012), and two independent Bayesian phylogenetic analyses were performed with each dataset under strict molecular clock, 100 000 000 generations, gamma substitution rates and rate heterogeneity among sites. After the removal of the initial 10% generations as *burn‐in*, the quality of the runs were assessed using tracer (http://beast.bio.ed.ac.uk/Tracer), regarding the convergence of likelihood values. Finally, the results of the independent runs (mitochondrial and nuclear datasets) were combined using logcombiner (beast2 package), and the *16S *+ *CO1* and *18S* rRNA maximum clade credibility (MCC) trees were generated with treeannotator (beast2 package). The final trees were then produced and edited with figtree (http://tree.bio.ed.ac.uk/software/figtree/).

**Figure 2:**
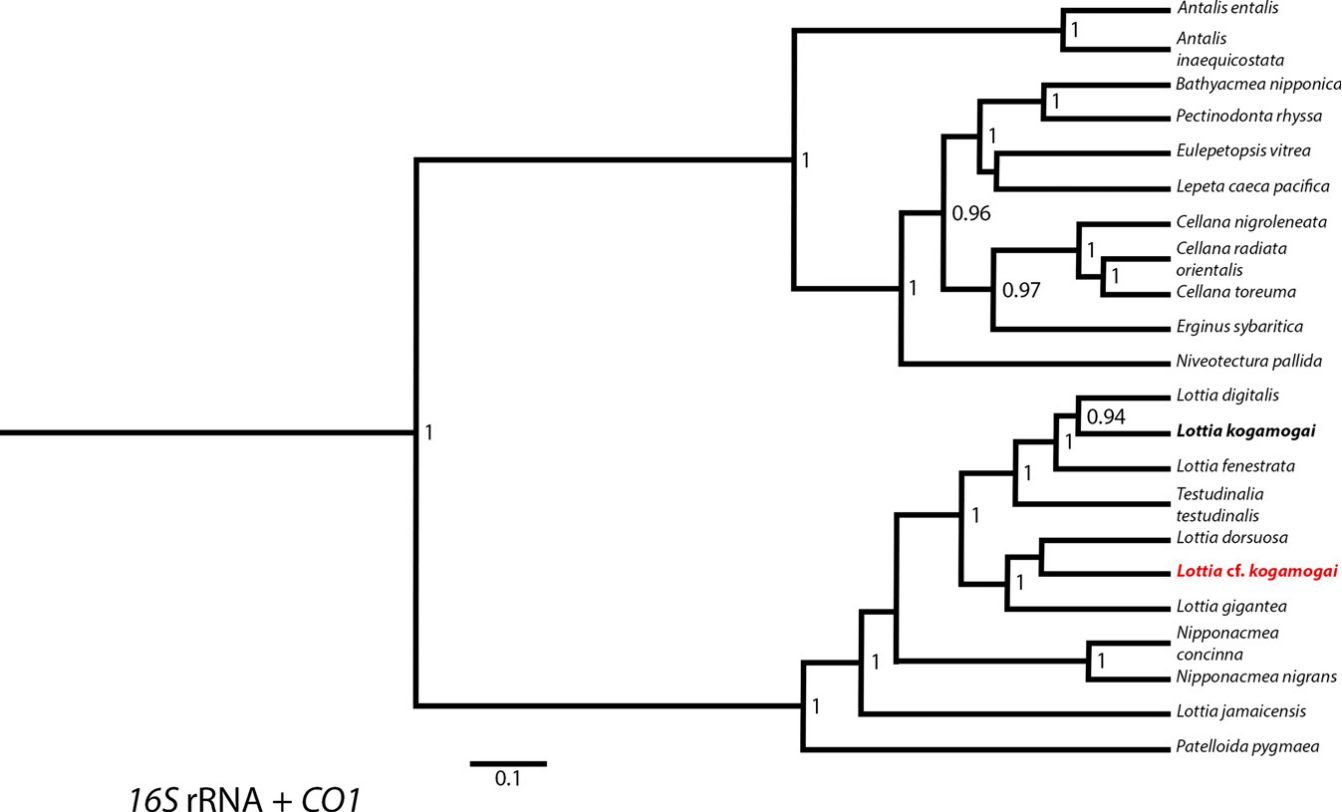
Maximum clade credibility (MCC) tree based on the two mitochondrial genes *16S* rRNA and *cytochrome c oxidase* subunit 1 (*CO1*) resulting from beast2 Bayesian analysis. Support values are posterior probabilities. Posterior probabilities < 0.9 are not shown. The species investigated in this study, and for which novel sequence data were generated, is indicated in red letters. Outgroup: *Antalis entalis* (Linnaeus, 1758), *Antalis inaequicostata* (Dautzenberg, 1891)

Both maximum clade credibility trees, using the genes *16S *+ *CO1* and *18S*, show that *L*. cf. *kogamogai* clusters together with other members of Lottiidae (Figs 2 and 3), despite the low support values in the deeper nodes of the *18S* phylogeny (Fig. 3). The combined mitochondrial (*16S *+ *CO1*) analysis shows with high support values of posterior probability that *L. *cf. *kogamogai* is not most closely related to *L. kogamogai* (formerly a member of *Collisella heroldi* complex; Fig. 2). Instead, *L*. *kogamogai* shows a sister group relationship with *Lottia digitalis* (Rathke, 1833), whereas *L. *cf. *kogamogai* is located outside that clade of lottiids, which is also corroborated by the genetic distance (number and proportion of base substitutions per site) between these sequences (Fig. 2; Tables S2 and S3). A close sister group relationship of *L*. cf. *kogamogai* to *L*. *digitalis* is also not recovered in the nuclear (*18S*) single gene analyses (Fig. 3). Our DNA sequence data suggest a different and maybe new limpet species in the Sea of Japan.

**Figure 3:**
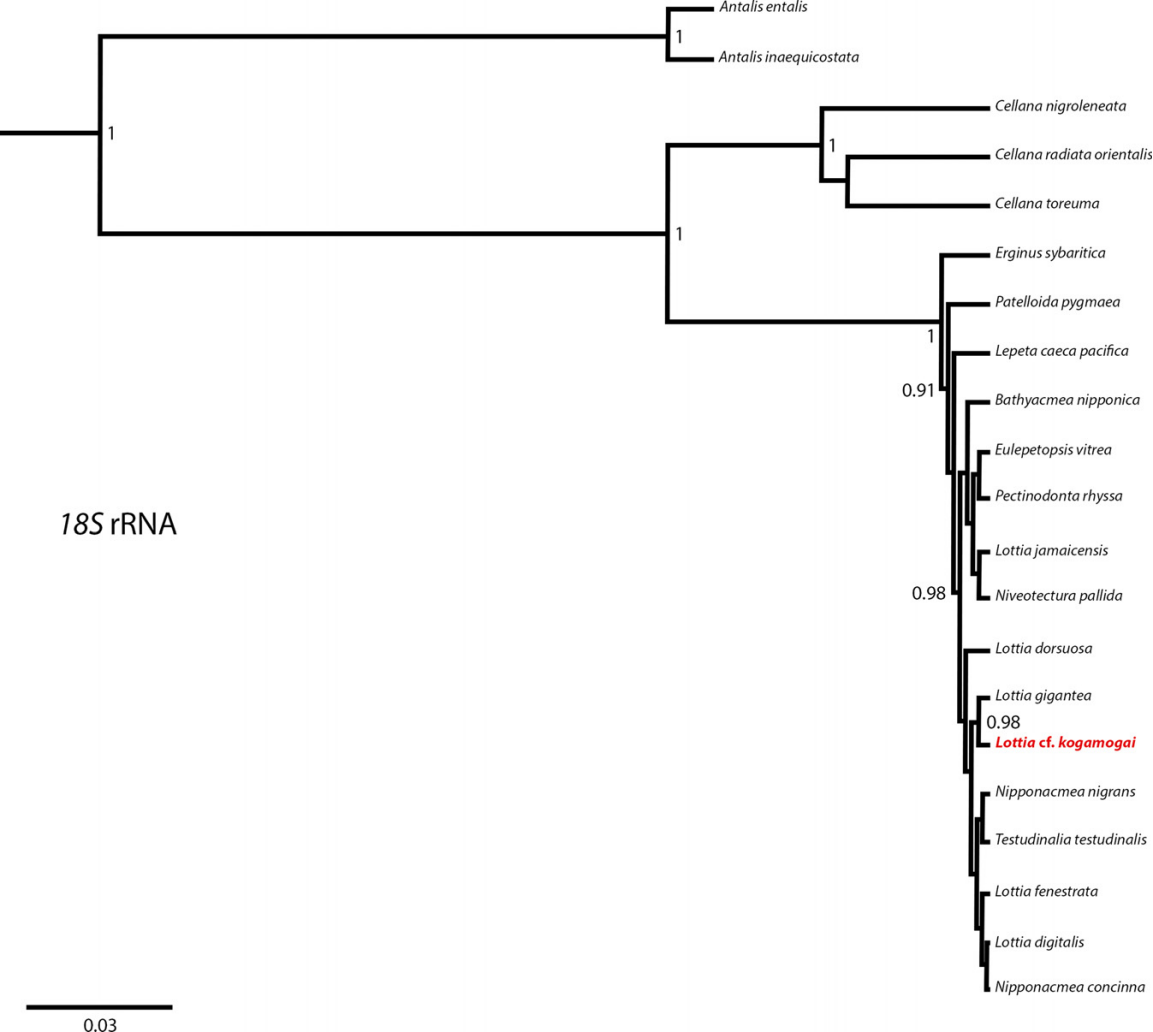
Maximum clade credibility (MCC) tree based on the nuclear gene *18S* rRNA resulting from beast2 Bayesian analysis. Support values are posterior probabilities. Posterior probabilities < 0.9 are not shown. The species investigated in this study, and for which novel sequence data were generated, is indicated in red letters. Outgroup: *Antalis entalis* (Linnaeus, 1758), *Antalis inaequicostata* (Dautzenberg, 1891). Note that since no *18S* sequence is publicly available, *Lottia kogamogai* Sasaki and Okutani 1994, is missing in this analysis.

Populations of *L. kogamogai* are reported to inhabit predominantly intertidal rocky shores from the eastern coast of the Japanese islands to Taiwan (Sasaki and Okutani 1994; Niu et al. 1998; Suzuki et al. 2010). The same patellogastropod species is reported to commonly inhabit also the Russian coasts of the Sea of Japan (Chernyshev and Chernova 2007; Gulbin 2010). The mitochondrial sequences of the patellogastropod species *Lottia* cf. *kogamogai* collected in the Vostok Bay (Sea of Japan, Russian Federation) and investigated herein are different from that published for *L. kogamogai* (Nakano and Ozawa 2004, 2007). Although the outer morphology of that Russian limpet appears very similar to the description of the Japanese *L*. *kogamogai*, the phylogenetic analyses provided herein indicate that these two limpets are different species (Figs 2 and 3). This discrepancy between the morphological (e.g. shell shape, colour, ornamentation) and molecular data highlights the intraspecific variation in patellogastropods, a phenomenon more common than previously thought (Simison and Lindberg 1999; Nakano and Ozawa 2005). The results also show that the phylogeny based on the nuclear ribosomal *18S* gene is not robust (posterior probability < 0.9) for a number of nodes within the patellogastropods. This is possibly due to the widely different substitution rates among these species, as suggested by Harasewych and McArthur (2000) and Nakano and Ozawa (2007), and difficulties in the alignment of the *18S* gene (long insertions and deletions). By contrast, the use of two different genes (*16S *+ *CO1*) with a more uniform evolutionary rate proved to be more informative concerning patellogastropod inter‐relationships.

Nakano and Sasaki (2011) noted in their review about Japanese limpets that several species including *L. kogamogai* are geographically and/or ecologically isolated, rendering them true biological species. Regional hydrographic conditions such as water currents, circulation, salinity and temperature are known factors that might act as geographical barriers that separate species (Yu et al. 2014). In general, the Sea of Japan contains cold water currents coming from the northern subpolar zones that run southwards along the Russian and Korean coasts, and relatively warm currents that come from the southern subtropical zones and run northwards along the Japanese archipelago (Fig. 1b; Moors et al. 2006). Although a water flow (circulation) among these cold and warm currents is present, regional hydrographic conditions are known from a number of areas, including the Peter the Great Bay, which might act as barrier for species distribution.

It remains to be shown by further molecular genetic and morphological studies whether all patellogastropod limpets that appear as *L. kogamogai* along the Russian east coast are one (or even more) different species as shown herein or if this is only true for the population in the Vostok Bay. Whether or not *L*. *kogamogai* represents a species complex in these waters as indicated by these molecular genetic results remains to be shown in future studies. However, our study provides sequences of three genes with which these limpets (i.e. *L*. cf. *kogamogai*) from Vostok Bay can now easily be identified. Furthermore, this discrepancy between the morphological and molecular genetic characters might also be true for numerous other patellogastropods such as *Limalepeta lima* (Dall, 1918), *Niveotectura pallida* (Gould, 1859) and *Lottia tenuisculpta* that are reported to be distributed in Japanese and Russian Far East waters as well (Sasaki 1998; Chernyshev and Chernova 2007; Gulbin 2010). Thus, it appears about time for a revision of these species by an integrative approach using molecular genetics as well as morphological characters.

## Supporting information


**Table S1.** List of species and genes with GenBank accession numbers used for phylogenetic analyses.
**Table S2.** Phenotypic distances (sequence divergence) between *16S* rRNA and *CO1*.
**Table S3.** Estimates of evolutionary divergence (base substitutions per site) between *16S* rRNA and *CO1* using the Jukes–Cantor model.
**Figure S1.** Maximum clade credibility (MCC) trees using individual *16S* rRNA and *CO1* mitochondrial markers resulting from BEAST2 Bayesian analysis.
