# Supplementary material for: A putative species complex in the Sea of Japan revealed by DNA sequence data: a study on Lottia cf. kogamogai (Gastropoda: Patellogastropoda)
Source: J Zool Syst Evol Res. 2016 Jan 8;54(3):177–81. doi: 10.1111/jzs.12120 (PMC4936521; doi:10.1111/jzs.12120)
Supplement: Supplementary file 1 — Table S1. List of species and genes with GenBank accession numbers used for phylogenetic analyses. Table S2. Phenotypic distances (sequence divergence) between 16S rRNA and CO1. Table S3. Estimates of evolutionary divergence (base substitutions per site) between 16S rRNA and CO1 using the Jukes–Cantor model. Figure S1. Maximum clade credibility (MCC) trees using individual 16S rRNA and CO1 mitochondrial markers resulting from BEAST2 Bayesian analysis. [file JZS-54-177-s001.pdf]

Supplementary Information

[1] *Department of Integrative Zoology, University of Vienna, UZA1 Althanstrasse 14, 1090 Vienna, Austria*

[2]*Laboratory of Cell Differentiation, A.V. Zhirmunsky Institute for Marine Biology, Far East Branch of the Russian Academy of Sciences, Paltchevsky ulica 17, 690041 Vladivostok, Russian Federation*

A putative species complex in the Sea of Japan revealed by DNA sequence data: A study on *Lottia* cf. *kogamogai* (Gastropoda: Patellogastropoda)

ALEN KRISTOF[1*], ANDRÉ LUIZ DE OLIVEIRA[1], KONSTANTIN G. KOLBIN[2] & ANDREAS WANNINGER[1]

**Corresponding author*: alen.kristof@univie.ac.at

Contributing authors: andre.luiz.de.oliveira@univie.ac.at; konstantin.kolbin@gmail.com; andreas.wanninger@univie.ac.at

Table S1. List of species and genes with GenBank accession numbers used for phylogenetic analyses

**Clade**/Family	**Species**	***16S***	***CO1***	***18S***
**Patellogastropoda**				
Nacellidae	*Cellana nigrolineata*	DQ093467	AB548213	DQ013353
	Cellana radiata orientalis	AB106478	AB433647	AB282768
	Cellana toreuma	GQ455937	JQ313557	AF308646
Lepetidae	*Lepeta caeca pacifica*	AB238347	AB543978	AB282759
Pectinodontidae	*Bathyacmaea nipponica*	AB238451	AB238588	AB282772
	Pectinodonta rhyssa	AB238452	AB238589	AB282773
Lottiidae	*Erginus sybaritica*	AB238350	AB238461	AB282761
	Lottia digitalis	AB238352	KF643845	DQ248942
	Lottia dorsuosa	AB106502	KM221054	AF308645
	Lottia fenestrata	FJ977695	FJ977749	FJ977631
	Lottia gigantea	FJ977696	FJ977750	FJ977632
	Lottia jamaicensis	FJ977697	FJ977751	FJ977633
	Lottia kogamogai	AB106493	AB238467	-
	Lottia* cf. *kogamogai	KU053948	KU053950	KU053949
	Nipponacmea concinna	AB106511	KM221077	DQ013354
	Nipponacmea nigrans	AB106516	AB238490	AB282763
	Niveotectura pallida	AB106519	AB238494	AF308644
	Testudinalia testudinalis	FJ977694	FJ977748	FJ977630
Neolepetopsidae	*Eulepetopsis vitrea*	DQ093468	DQ093516	DQ093427
Patelloidinae	*Patelloida pygmaea*	AB161514	AB196505	AB282765
Outgroup				
**Scaphopoda**				
Dentaliidae	*Antalis entalis*	DQ280027	DQ280016	DQ279936
	Antalis inaequicostata	DQ280026	DQ280015	DQ279935

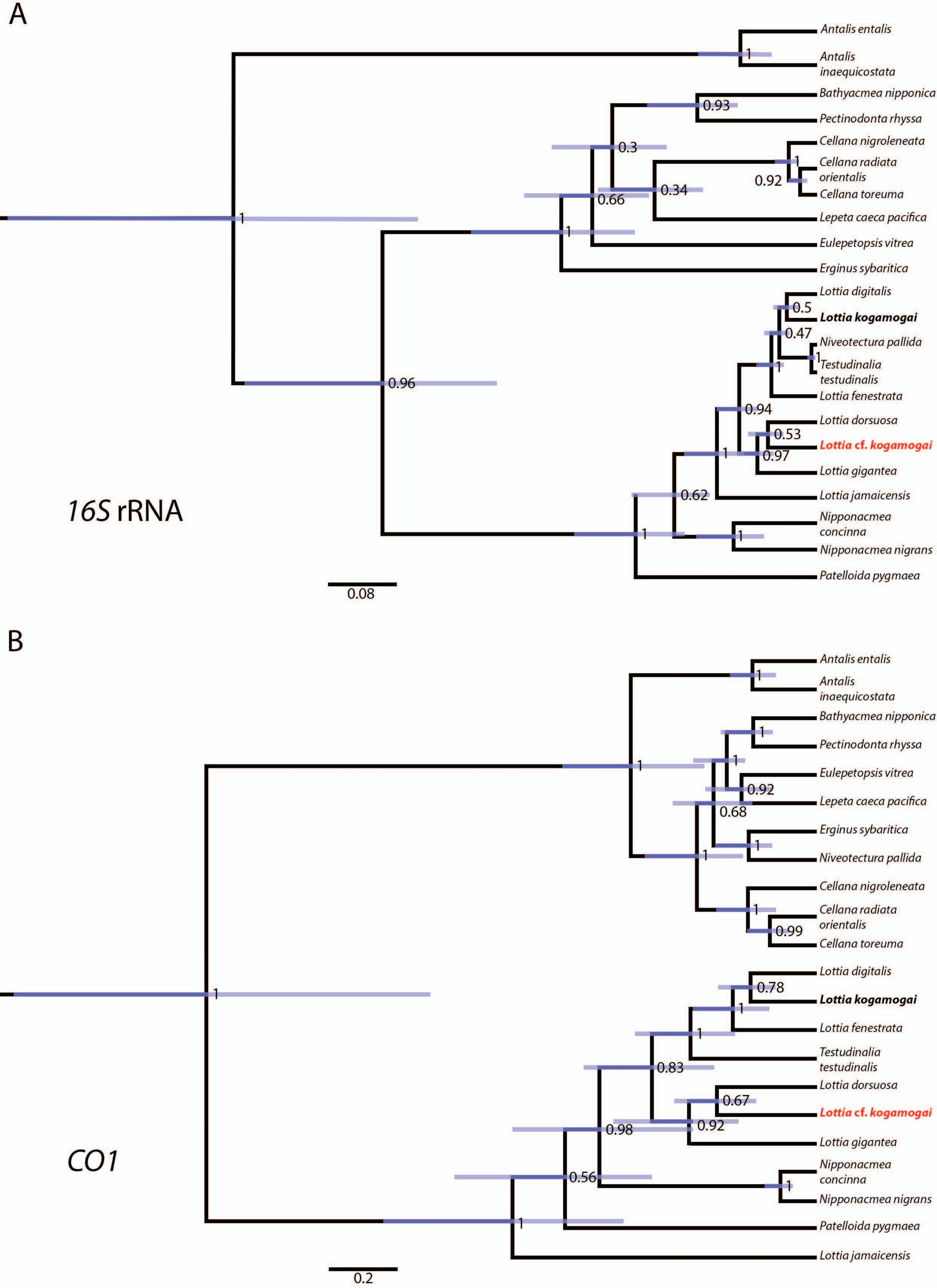
A
Antalis entalis
Antalis inaequicostata
Bathyacmea nipponica
Pectinodonta rhyssa
Cellana nigroleneata
Cellana radiata orientalis
Cellana toreuma
Lepeta caeca pacifica
Eulepetopsis vitrea
Erginus sybaritica
Lottia digitalis
Lottia kogamogai
Niveotectura pallida
Testudinalia testudinalis
Lottia fenestrata
Lottia dorsuosa
Lottia cf. kogamogai
Lottia gigantea
Lottia jamaicensis
Nipponacmea concinna
Nipponacmea nigrans
Patelloida pygmaea
1
0.93
0.3
0.92
0.34
0.66
0.96
0.5
0.47
0.94
0.53
0.97
0.62
16S rRNA
0.08
B
Antalis entalis
Antalis inaequicostata
Bathyacmea nipponica
Pectinodonta rhyssa
Eulepetopsis vitrea
Lepeta caeca pacifica
Erginus sybaritica
Niveotectura pallida
Cellana nigroleneata
Cellana radiata orientalis
Cellana toreuma
Lottia digitalis
Lottia kogamogai
Lottia fenestrata
Testudinalia testudinalis
Lottia dorsuosa
Lottia cf. kogamogai
Lottia gigantea
Nipponacmea concinna
Nipponacmea nigrans
Patelloida pygmaea
Lottia jamaicensis
0.92
0.68
0.99
0.78
0.83
0.67
0.92
0.98
0.56
CO1
0.2

Figure S1. Maximum clade credibility (MCC) trees using individual *16S* rRNA and *cytochrome c oxidase* subunit I (*CO1*) mitochondrial markers resulting from BEAST2 Bayesian analysis. *Lottia* cf. *kogamogai* is indicated in red and *Lottia kogamogai* Sasaki and Okutani, 1994 in bold letters. All clades are shown with their associated 95% confidence intervals (blue bars). The support values are posterior probabilities. Outgroup: *Antalis entalis* (Linnaeus, 1758), *Antalis inaequicostata* (Dautzenberg, 1891). **A.** MCC tree based on the *16S* gene. **B.** MCC tree based on the *CO1* gene.

Table S2. Phenotypic distances (sequence divergence) between *16S* rRNA and *cytochrome c oxidase* subunit 1 (*CO1*). Proportion of base substitutions per site between *16S* (above the diagonal) and *CO1* (below the diagonal) sequences are shown. The analysis involved 22 nucleotide sequences. All positions containing gaps and missing data were eliminated. There are a total of 248 (*16S*) and 548 (*CO1*) positions in the final dataset. Calculation performed in MEGA 6 [1].

	1	2	3	4	5	6	7	8	9	10	11	12	13	14	15	16	17	18	19	20	21	22
Antalis entalis (1)	-	0.150	0.315	0.335	0.339	0.347	0.351	0.355	0.331	0.399	0.403	0.399	0.379	0.411	0.403	0.403	0.379	0.379	0.403	0.367	0.335	0.395
Antalis inaequicostata (2)	0.171	-	0.302	0.306	0.290	0.294	0.327	0.355	0.306	0.395	0.411	0.403	0.387	0.419	0.391	0.399	0.383	0.379	0.403	0.351	0.310	0.395
Bathyacmaea nipponica (3)	0.262	0.297	-	0.262	0.25	0.254	0.254	0.234	0.246	0.355	0.347	0.355	0.343	0.391	0.351	0.347	0.355	0.331	0.347	0.323	0.218	0.347
Cellana nigrolineata (4)	0.297	0.301	0.234	-	0.0726	0.077	0.222	0.234	0.214	0.319	0.315	0.310	0.315	0.339	0.331	0.323	0.335	0.323	0.327	0.315	0.198	0.323
Cellana radiata orientalis (5)	0.294	0.301	0.234	0.126	-	0.052	0.214	0.230	0.206	0.302	0.306	0.302	0.290	0.319	0.319	0.302	0.315	0.306	0.315	0.302	0.169	0.306
Cellana toreuma (6)	0.308	0.332	0.28	0.178	0.157	-	0.222	0.226	0.222	0.302	0.306	0.306	0.302	0.335	0.331	0.306	0.319	0.310	0.315	0.290	0.198	0.302
Erginus sybaritica (7)	0.262	0.276	0.185	0.161	0.175	0.224	-	0.242	0.226	0.290	0.282	0.282	0.298	0.319	0.302	0.286	0.298	0.286	0.302	0.310	0.242	0.302
Eulepetopsis vitrea (8)	0.287	0.266	0.206	0.22	0.238	0.231	0.192	-	0.262	0.282	0.274	0.298	0.258	0.282	0.278	0.274	0.286	0.266	0.302	0.290	0.266	0.298
Lepeta caeca pacifica (9)	0.273	0.294	0.192	0.21	0.224	0.224	0.185	0.15	-	0.343	0.331	0.335	0.343	0.363	0.339	0.339	0.310	0.327	0.359	0.315	0.206	0.351
Lottia digitalis (10)	0.413	0.381	0.43	0.381	0.413	0.427	0.406	0.381	0.395	-	0.097	0.065	0.109	0.149	0.093	0.028	0.173	0.153	0.073	0.198	0.347	0.069
Lottia dorsuosa (11)	0.381	0.385	0.413	0.374	0.402	0.392	0.374	0.385	0.371	0.224	-	0.121	0.101	0.153	0.089	0.081	0.173	0.157	0.121	0.206	0.335	0.113
Lottia fenestrata (12)	0.406	0.392	0.434	0.406	0.399	0.413	0.406	0.381	0.392	0.213	0.287	-	0.121	0.149	0.109	0.056	0.165	0.145	0.089	0.206	0.335	0.089
Lottia gigantea (13)	0.402	0.399	0.406	0.374	0.406	0.406	0.381	0.406	0.395	0.238	0.203	0.28	-	0.145	0.105	0.101	0.157	0.157	0.117	0.198	0.323	0.105
Lottia jamaicensis (14)	0.374	0.402	0.451	0.399	0.402	0.402	0.423	0.409	0.413	0.332	0.325	0.315	0.315	-	0.133	0.145	0.181	0.198	0.161	0.218	0.371	0.153
Lottia cf. *kogamogai* (15)	0.364	0.378	0.395	0.378	0.409	0.399	0.388	0.364	0.388	0.276	0.217	0.301	0.213	0.329	-	**0.085**	0.173	0.161	0.109	0.198	0.339	0.117
Lottia kogamogai (16)	0.43	0.441	0.451	0.434	0.434	0.444	0.434	0.441	0.43	0.199	0.276	0.227	0.28	0.322	**0.311**	-	0.169	0.149	0.077	0.202	0.343	0.073
Nipponacmea concinna (17)	0.381	0.399	0.385	0.381	0.409	0.43	0.392	0.399	0.402	0.283	0.245	0.318	0.22	0.357	0.22	0.301	-	0.141	0.173	0.222	0.323	0.165
Nipponacmea nigrans (18)	0.399	0.406	0.409	0.388	0.399	0.434	0.402	0.416	0.392	0.315	0.259	0.294	0.227	0.343	0.245	0.308	0.112	-	0.169	0.210	0.323	0.165
Niveotectura pallida (19)	0.294	0.311	0.227	0.203	0.224	0.203	0.154	0.21	0.22	0.409	0.364	0.434	0.374	0.399	0.367	0.437	0.413	0.409	-	0.202	0.339	0.02
Patelloida pygmaea (20)	0.395	0.402	0.399	0.406	0.409	0.416	0.395	0.406	0.402	0.332	0.308	0.336	0.28	0.325	0.304	0.35	0.325	0.318	0.406	-	0.319	0.19
Pectinodonta rhyssa (21)	0.276	0.283	0.168	0.231	0.217	0.255	0.196	0.206	0.175	0.406	0.378	0.399	0.392	0.423	0.392	0.434	0.378	0.381	0.213	0.392	-	0.335
Testudinalia testudinalis (22)	0.395	0.437	0.385	0.395	0.402	0.378	0.42	0.395	0.371	0.21	0.262	0.252	0.241	0.318	0.245	0.241	0.28	0.297	0.42	0.308	0.395	-

16S

CO1

[1] Tamura K, Stecher G, Peterson D, Filipski A, Kumar S (2013) MEGA6: Molecular evolutionary genetics analysis version 6.0. Mol Biol Evol **30:** 2725-2729.

Table S3. Estimates of evolutionary divergence using the Jukes-Cantor model [1] between *16S* rRNA and *cytochrome c oxidase* subunit 1 (*CO1*). The number of base substitutions per site between 16S (above the diagonal) and *CO1* (below the diagonal) sequences are shown. The analysis involved 22 nucleotide sequences. All positions containing gaps and missing data were eliminated. There are a total of 248 (*16S*) and 548 (*CO1*) positions in the final dataset. Evolutionary analyses were conducted in MEGA6 [2].

	1	2	3	4	5	6	7	8	9	10	11	12	13	14	15	16	17	18	19	20	21	22
Antalis entalis (1)	-	0.166	0.408	0.443	0.451	0.465	0.473	0.481	0.436	0.57	0.579	0.57	0.528	0.596	0.579	0.579	0.528	0.528	0.579	0.504	0.443	0.561
Antalis inaequicostata (2)	0.195	-	0.387	0.394	0.367	0.374	0.429	0.481	0.394	0.561	0.596	0.579	0.544	0.614	0.553	0.57	0.536	0.528	0.579	0.473	0.401	0.561
Bathyacmaea nipponica (3)	0.323	0.378	-	0.322	0.304	0.31	0.31	0.28	0.298	0.481	0.465	0.481	0.458	0.553	0.473	0.465	0.481	0.436	0.465	0.422	0.257	0.465
Cellana nigrolineata (4)	0.378	0.384	0.281	-	0.076	0.081	0.263	0.28	0.252	0.415	0.408	0.401	0.408	0.451	0.436	0.422	0.443	0.422	0.429	0.408	0.229	0.422
Cellana radiata orientalis (5)	0.373	0.384	0.281	0.138	-	0.054	0.252	0.274	0.24	0.387	0.394	0.387	0.367	0.415	0.415	0.387	0.408	0.394	0.408	0.387	0.192	0.394
Cellana toreuma (6)	0.396	0.439	0.35	0.204	0.177	-	0.263	0.269	0.263	0.387	0.394	0.394	0.387	0.443	0.436	0.394	0.415	0.401	0.408	0.367	0.229	0.387
Erginus sybaritica (7)	0.323	0.345	0.213	0.181	0.199	0.266	-	0.292	0.269	0.367	0.354	0.354	0.38	0.415	0.387	0.361	0.38	0.361	0.387	0.401	0.292	0.387
Eulepetopsis vitrea (8)	0.361	0.328	0.241	0.261	0.286	0.276	0.222	-	0.322	0.354	0.341	0.38	0.316	0.354	0.348	0.341	0.361	0.329	0.387	0.367	0.329	0.38
Lepeta caeca pacifica (9)	0.339	0.373	0.222	0.246	0.266	0.266	0.213	0.168	-	0.458	0.436	0.443	0.458	0.496	0.451	0.451	0.401	0.429	0.488	0.408	0.24	0.473
Lottia digitalis (10)	0.599	0.532	0.639	0.532	0.599	0.631	0.584	0.532	0.561	-	0.104	0.067	0.118	0.166	0.099	0.029	0.197	0.171	0.076	0.229	0.465	0.072
Lottia dorsuosa (11)	0.532	0.539	0.599	0.518	0.576	0.554	0.518	0.539	0.511	0.266	-	0.132	0.108	0.171	0.094	0.085	0.197	0.176	0.132	0.24	0.443	0.122
Lottia fenestrata (12)	0.584	0.554	0.647	0.567	0.569	0.599	0.584	0.532	0.554	0.251	0.361	-	0.132	0.166	0.118	0.059	0.187	0.161	0.094	0.24	0.443	0.094
Lottia gigantea (13)	0.576	0.569	0.584	0.518	0.584	0.584	0.532	0.584	0.561	0.286	0.236	0.35	-	0.161	0.113	0.108	0.176	0.176	0.127	0.229	0.422	0.113
Lottia jamaicensis (14)	0.518	0.576	0.69	0.569	0.576	0.576	0.623	0.591	0.599	0.439	0.426	0.408	0.408	-	0.146	0.161	0.208	0.229	0.182	0.257	0.512	0.171
Lottia cf. *kogamogai* (15)	0.497	0.525	0.561	0.525	0.591	0.569	0.547	0.497	0.547	0.345	0.256	0.384	0.251	0.432	-	**0.09**	0.197	0.182	0.118	0.229	0.451	0.127
Lottia kogamogai (16)	0.639	0.664	0.69	0.647	0.647	0.673	0.647	0.664	0.639	0.232	0.345	0.271	0.35	0.42	**0.402**	-	0.192	0.166	0.081	0.235	0.458	0.076
Nipponacmea concinna (17)	0.532	0.569	0.539	0.532	0.591	0.639	0.554	0.569	0.576	0.356	0.296	0.414	0.261	0.484	0.261	0.384	-	0.156	0.197	0.263	0.422	0.187
Nipponacmea nigrans (18)	0.569	0.584	0.591	0.547	0.569	0.647	0.576	0.607	0.554	0.408	0.317	0.373	0.271	0.458	0.296	0.396	0.121	-	0.192	0.246	0.422	0.187
Niveotectura pallida (19)	0.373	0.402	0.271	0.236	0.266	0.236	0.172	0.246	0.261	0.591	0.497	0.647	0.518	0.569	0.504	0.656	0.599	0.591	-	0.235	0.451	0.02
Patelloida pygmaea (20)	0.561	0.576	0.569	0.584	0.591	0.607	0.561	0.584	0.576	0.439	0.396	0.445	0.35	0.426	0.39	0.471	0.426	0.414	0.584	-	0.415	0.218
Pectinodonta rhyssa (21)	0.345	0.356	0.19	0.276	0.256	0.312	0.227	0.241	0.199	0.584	0.525	0.569	0.554	0.623	0.554	0.647	0.525	0.532	0.251	0.554	-	0.443
Testudinalia testudinalis (22)	0.561	0.656	0.539	0.561	0.576	0.525	0.615	0.561	0.511	0.246	0.323	0.307	0.291	0.414	0.296	0.291	0.35	0.378	0.615	0.396	0.561	-

16S
CO1

[1] Jukes TH, Cantor CR (1969) Evolution of protein molecules. In: Munro HN (ed), Mammalian Protein Metabolism. Academic Press, New York, pp 21-132.

[2] Tamura K, Stecher G, Peterson D, Filipski A, Kumar S (2013) MEGA6: Molecular evolutionary genetics analysis version 6.0. Mol Biol Evol **30:** 2725-2729.
